# Exploring the effect of nsSNPs in human YPEL3 gene in cellular senescence

**DOI:** 10.1038/s41598-020-72333-8

**Published:** 2020-09-17

**Authors:** Abhishek Singh, Mukesh Thakur, Sujeet Kumar Singh, Lalit Kumar Sharma, Kailash Chandra

**Affiliations:** Zoological Survey of India, New Alipore, Kolkata, 700053 India

**Keywords:** Cancer genetics, Computational biology and bioinformatics

## Abstract

YPEL3 that induces cellular senescence in both normal and tumour cells of humans may show altered expression under the influence of incidental mutations. In this study, we proposed the first structure of Native YPEL3 protein and its five possible deleterious mutants—V40M, C61Y, G98R, G108S, and A131T and predicted their deleterious effects to alter stability, flexibility and conformational changes in the protein. The MD simulation (RMSD, RMSF, Rg, h-bond and SASA) analysis revealed that the variants V40M, G98R and G108S increased the flexibility in protein, and variant V40M imparted more compactness to the protein.. In general, variants attributed changes in the native conformation and structure of the YPEL3 protein which might affect the native function of cellular senescence. The study provides opportunities for health professionals and practitioners in formulating précised medicines to effectively cure various cancers. We propose in-vitro or in-vivo studies should consider these reported nsSNPs while examining any malfunction in the YPEL3 protein.

## Introduction

Single nucleotide polymorphisms (SNPs) are the most common type of genetic variation contributing about 90% of the total human genome polymorphism^1^. Identification of these variations and their impact on human health is one of the least explored areas in the field of human genetics. Around 0.12% of genetic variants are predicted deleterious to human health^1^. These genetic variations, in particular nonsynonymous SNPs (nsSNPs) caused by mutations may affect the structure or function of the protein, by changing the amino acids. However, not all nsSNPs cause damages to the protein. Many of them show disease phenotype and a few of them are neutral^2^. Therefore, appropriate selection of bio-computational methods, in distinguishing the deleterious nsSNPs from the neutral ones is prerequisite towards predicting the structural and functional consequences of the target proteins. A few earlier studies investigated the pathogenic effects of nsSNPs on the structure and function of various native protein^3–7^, and also identified drug resistance mechanism due to point mutations in *Mycobacterium tuberculosis*^8^. Rajendran et al.^4^ investigated that the alterations caused due to the point mutation, P29S in RAC1, increased the rate of cell proliferation leading to a cancerous state. These studies have signified the imperativeness of computational investigations in elucidating the role of nsSNPs and laid foundation to rely on the in-silico analysis to predict cellular mechanism responsible for various genetic disorders. In the present study, we tried to address how the predicted genetic variants affect the structure and functionality of the 'Yippee like 3′ (YPEL3) protein in humans. This protein is encoded by YPEL3 gene which is a member of five closely related paralogues—YPEL1-5 which are named in reference to their Drosophila orthologue^9^. Baker^10^ described YPEL3 as a small unstable apoptotic protein (SUAP), which stimulated the removal of IL3 from the myelenoid precursor cell line leading to apoptosis. In addition to this, YPEL3 was also found to be degraded by proteosomes describing it as unstable protein^10^. The Human YPEL3 gene is located on the short arm of chromosome 16 (16p11.2), having high sequence conservation in wide range of species. In humans, two functional transcript variants of YPEL3 have been identified, i.e. Transcript variant 1 (GeneBank ID: NM_031477.4) encodes for 157 amino acid residue protein of approximately 17.5 kDa. The other transcript variant 2 (GeneBank ID: NM_001145524.1) encodes for a 119 amino acid protein of approximately 13.6 kDa. The transcript variant 1 has an additional 29 amino acids compared to transcript variant 2 at the N terminus. Further, YPEL3 is reported to be a p53 target gene showing growth-suppressive properties like senescence and apoptosis under various circumstances^11^. Due to inadequate information about the structure and the impact of variants in YPEL3 protein, limited attempts are made to explore the précised medicines facilitating cure for several cancers in the context of mutation driven alteration in the structure and function of the protein. In this study, we determined the structural and functional impact of most deleterious nsSNPs of YPEL3 protein by using the computational simulations and proposed three dimensional structures of YPEL3 protein and its mutants. We also applied several tools to prioritize damaging nsSNPs and assessed the most deleterious one based on stability assessment, evolutionary conservation analysis and post translational modification site prediction. We modelled the 3D structure of both native and mutant YPEL3 protein and analyzed the conformational behavior based on simulations. We believe this study will aid to the ongoing research on cancer genetics and seeking for therapeutic candidates.

## Results

### Mining of nsSNPs in YPEL3 gene

Of the total 1528 SNPs reported in dbSNP database for YPEL3 gene (359 UTR variants, 861 intron variants and several others), 73 missense and 2 nonsense SNPs were mapped in Humans along with their meta information like alleles, chromosomal location and MAF (global minor allele frequency).

### Prediction of deleterious nsSNPs using PredictSNP2

A total 28 nsSNPs were found consentaneously deleterious with an expected accuracy range (EAR) of 0.87 to 0.72 by the five prediction tools. The tool CADD predicted 59 nsSNPs were deleterious, followed by 50 deleterious nsSNPs by GWAVA, 38 deleterious nsSNPs by DANN, 32 deleterious nsSNPs by FATHMM and only one deleterious nsSNPs was predicted by FunSeq. (Table S1).

### Prediction of deleterious nsSNPs using PredictSNP1

A total 10 nsSNPs i.e. V40M, R57L, C61Y, G98R, G108S, D114N, E129Q, A131T, A131V and I145T were found consentaneously deleterious by the seven prediction tools. Tool MAPP and PhD-SNP both predicted nine deleterious mutations, while the tool PolyPhen 1 and PolyPhen-2 predicted eight and 13 deleterious mutations. Similarly, SIFT predicted 13 deleterious mutations and nine deleterious mutations were predicted by SNAP. Overall 10 deleterious mutations were predicted by PredictSNP 1 (Table 1).

**Table 1:** PredictSNP1 result of 13 non-synonymous mutations in YPEL3 gene.

Mutation	SNP ID	PredictSNP	Confidence score (%)	MAPP	Confidence score (%)	PhD-SNP	Confidence score (%)	PolyPhen-1	Confidence score (%)	PolyPhen-2	Confidence score (%)	SIFT	Confidence score (%)	SNAP	Confidence score (%)
V40M	rs759413482	Deleterious	64	–	–	Neutral	68	Deleterious	74	Deleterious	47	Deleterious	53	Deleterious	72
R57L	rs1378953136	Deleterious	65	Deleterious	48	Deleterious	86	Neutral	67	Deleterious	40	Deleterious	46	Deleterious	72
C61Y	rs753385457	Deleterious	87	Deleterious	77	Deleterious	89	Deleterious	74	Deleterious	65	Deleterious	53	Deleterious	87
D73N	rs568854299	Neutral	65	Neutral	66	Neutral	58	Neutral	67	Deleterious	47	Deleterious	45	Neutral	61
G98R	rs1234813494	Deleterious	87	Deleterious	88	Deleterious	77	Deleterious	74	Deleterious	65	Deleterious	79	Deleterious	81
G108S	rs760745635	Deleterious	87	Deleterious	57	Deleterious	88	Deleterious	74	Deleterious	81	Deleterious	79	Deleterious	56
D114N	rs936261369	Deleterious	87	Deleterious	77	Deleterious	86	Deleterious	59	Deleterious	81	Deleterious	53	Deleterious	72
E129Q	rs1316376447	Deleterious	61	Neutral	64	Deleterious	86	Deleterious	59	Deleterious	55	Deleterious	53	Neutral	55
A131T	rs1295542567	Deleterious	72	Deleterious	78	Deleterious	88	Neutral	67	Deleterious	81	Deleterious	45	Deleterious	62
A131V	rs373399618	Deleterious	87	Deleterious	77	Deleterious	88	Deleterious	59	Deleterious	81	Deleterious	53	Deleterious	72
I145T	rs757003466	Deleterious	87	Deleterious	78	Deleterious	73	Deleterious	74	Deleterious	65	Deleterious	79	Deleterious	62
G155A	rs752040143	Neutral	60	Deleterious	41	Neutral	51	Neutral	67	Deleterious	45	Deleterious	43	Neutral	50
D157N	rs767025068	Neutral	63	–	–	Neutral	68	Neutral	67	Deleterious	45	Deleterious	45	Neutral	55

### Prediction of protein stability change and evolutionary conservation analysis

All mutations caused decrease in stability of protein based on the prediction of I-Mutant and MuPro except the mutation R57L (Table 2). The conservation score for each amino acid residue and their structural conformation being exposed or buried were predicted (Figure S1). Of 10 mutations predicted by PredictSNP1, six residues were structural and one was functional. The structural residues were present in the highly conserved region with a conservation score of nine. The residue R57 and I145 were predicted to be moderately conserved with a conservation score of seven and six (Table S2).

**Table 2:** Prediction of protein stability change due to nsSNPs in YPEL3 gene.

Mutations	I-Mutant			MuPro	
	DDG value	RI	Stability	ΔΔG	Stability
V40M	− 2.47	7	Decrease	− 1.047	Decreases
R57L	− 0.10	5	Decrease	0.363	Increases
C61Y	0.06	2	Decrease	− 0.854	Decreases
G98R	− 1.32	8	Decrease	− 0.165	Decreases
G108S	− 1.22	8	Decrease	− 1.001	Decreases
D114N	0.84	2	Decrease	− 0.954	Decreases
E129G	− 1.52	7	Decrease	− 1.174	Decreases
A131T	− 0.54	5	Decrease	− 0.950	Decreases
A131V	− 0.40	1	Decrease	− 0.391	Decreases
I145T	− 0.61	3	Decrease	− 2.272	Decreases

### Post-translational modification (PTM) site prediction

#### Methylation, phosphorylation, and ubiquitylation

GPS-MSP did not predict any Lysine and Arginine residue which may facilitate methylation. PMeS predicted a total of five arginine residues that may be methylated but no occurrence of lysine residue (Table S3). GPS 5.0 server predicted a total of 15 residues of which six were tyrosine specific sites, five for threonine-specific and 10 for serine specific phosphorylation sites. NetPhos 3.1 predicted seven serine specific sites, two each for threonine and tyrosine specific sites to facilitate phosphorylation (Table S4). All together, 11 common residue sites were predicted by both GPS 5.0 and NetPhos 3.1.

BDM-PUB predicted three lysine residues which may facilitate Ubiquitylation, while UbPred predicted only one lysine residue with a very low confidence score of 0.66, which may facilitate Ubiquitylation (Table S5).

### Prediction of three dimensional structures

**Figure 1:**
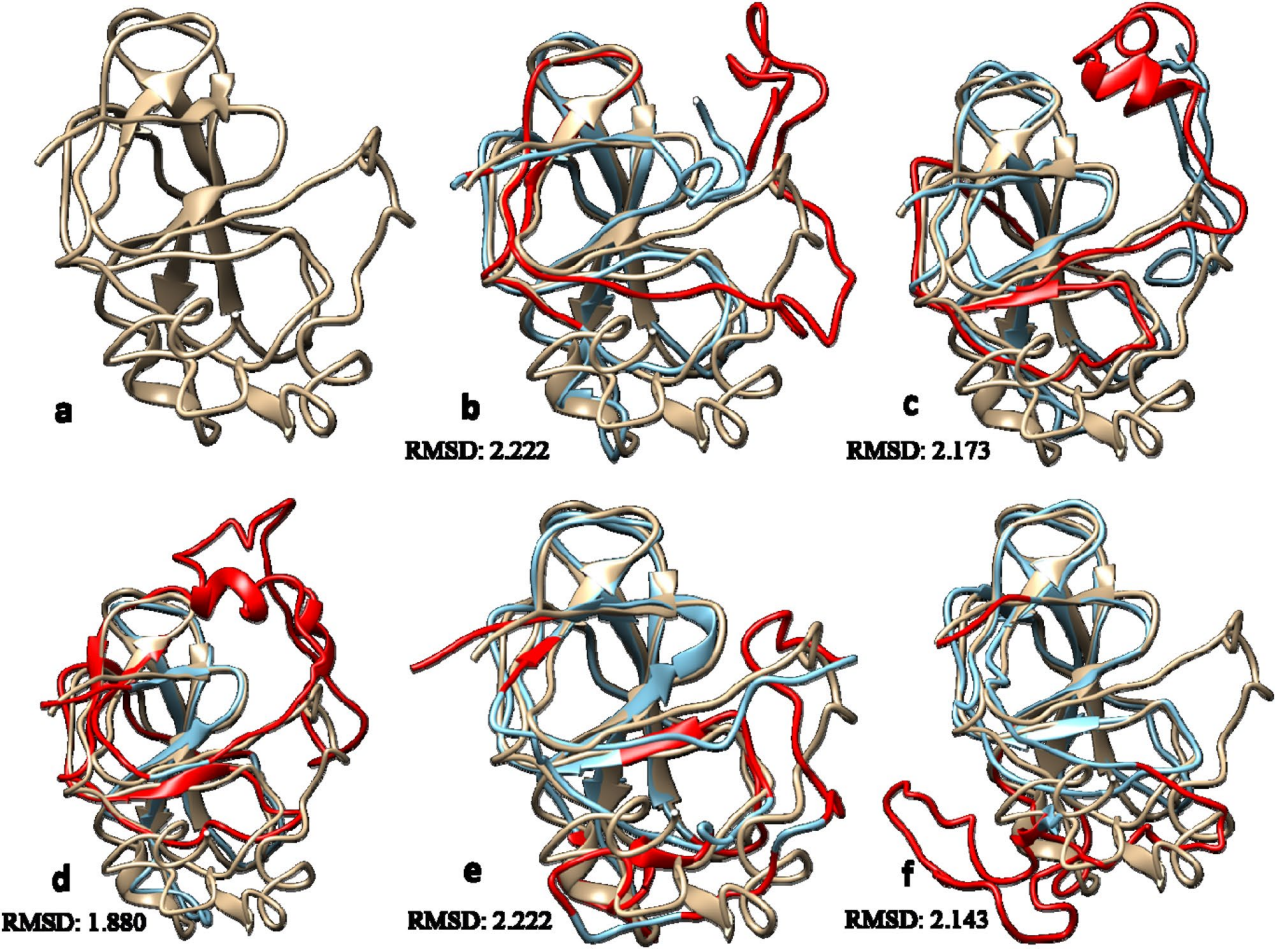
Predicted 3D structure of Native YPEL3 protein (light brown) superimposed by the different mutations (blue) with their respective RMSD values. The red colour denotes most deviated part of the mutated protein structure based on RMSF values **(a)**. Native YPEL3; **(b)**.V40M YPEL3; **(c)** G108S YPEL3; **(d)** G98R YPEL3; **(e)** C61Y YPEL3 and **(f)** A131T YPEL3.

The predicted model for the mutant I145T showed high deviation with an RMSD value of 2.514 Å, followed by V40M (RMSD = 2.222 Å), C61Y (RMSD = 2.222 Å), G108S (RMSD = 2.173 Å), A131T (RMSD = 2.143 Å) and G98R (RMSD = 1.880 Å). While mutants- R57L, D114N, A131V, and E129G did not show any variation with the native structure (Table S6). The top five models for both native type and each of the mutant proteins were then predicted using the I TASSER. From the mutant models that demonstrated a relatively high RMSD values i.e. V40M, C61Y, G108S, A131T, and G98R, the models with maximum ERRAT values and significant C-Score i.e. V40M (78.52-ERRAT; -2.82-C-Score), C61Y (79.86-ERRAT; -2.28- C-Score), G98R (83.89-ERRAT; -2.87- C-Score), G108S (76.51-ERRAT; -2.82- C-Score ) and A131T (75.83-ERRAT; -2.66- C-Score) (Table S7) were selected for superimposing over native model using Chimera 1.10.1. The superimposed structures for each five selected mutant models are shown in Fig. 1a–f.

### Validation and reliability of modelled proteins

All the modelled structures of both wild and mutant proteins possessed Z-score significantly different from zero. Wild (− 2.73), V40M (− 3.41), G108S (− 2.15), G98R (− 1.71), C61Y (− 2.85) and A131T (− 3.07) indicating less chances of error and high quality of structures (Figure S2). Further, the residues in the disallowed region were in the range of 5.2% to 1.5% lesser than the threshold of 10% (Table S8).

### Effect of mutations on structural features

Five mutations i.e.V40M, C61Y, G98R, G108S and A131T introduced relatively bigger residue than the wild type. The introduction of the bigger size of amino acids might lead to bumps in protein structure whereas smaller residues might lead to loss of molecular interaction. The change in the charge of amino acids was predicted in G98R (neutral to positive). This may lead to a loss of interaction between amino acids of the protein or between protein and other molecules. Mutations i.e. C61Y, G98R and A131T, replaced the native residues by a lower hydrophobic residue which might result in the loss of hydrophobic function on the surface or in the core region of the protein. In mutation G98R and G108S, the torsion angles for the mutant residues were unusual which changed the conformation and the local structure of the protein.

### Effect of mutations on secondary structure

The secondary structural analysis revealed the number of residues participating in the formation of secondary structural elements like Turn, Coil, Strand, 3–10 helix, Pi-helix, Alpha helix and Bridges in the wild and mutant protein structures (Table S9). Mutations altered the distribution of secondary structure elements from the wild and thus imparted the change in conformation of the protein (Figure S3). Interestingly, the secondary structure element Pi-helix was observed only in the wild protein. Pi-helix, a rare occurring element, contributes characteristic structural features within the protein, and its absence in cases of mutants might play a considerable role in the structure and function of the protein.

### Functional interaction and association network

Out of the top 10 interacting genes predicted by STRING, the WD repeat-containing protein 26 (WDR26) gene showed highest combined score of 0.716 (Table S10) based on co-expression, experiments and text mining data. The WDR26 was predicted as the highly interactive gene to YPEL3 (Figure S4). However, the Gene MANIA showed YPEL3 physically interacted with only a single gene i.e. DOK7 and co-expressed with 19 genes (Figure S5). It shared the protein domains with 14 genes and co-localized with 6 genes (Figure S5 and Table S11). Both of these database predicted interaction of YPEL3 with its biological functional partners and hence any mutation in YPEL3 could affect the interaction and function of the protein.

### Molecular dynamic (MD) simulation

**Figure 2:**
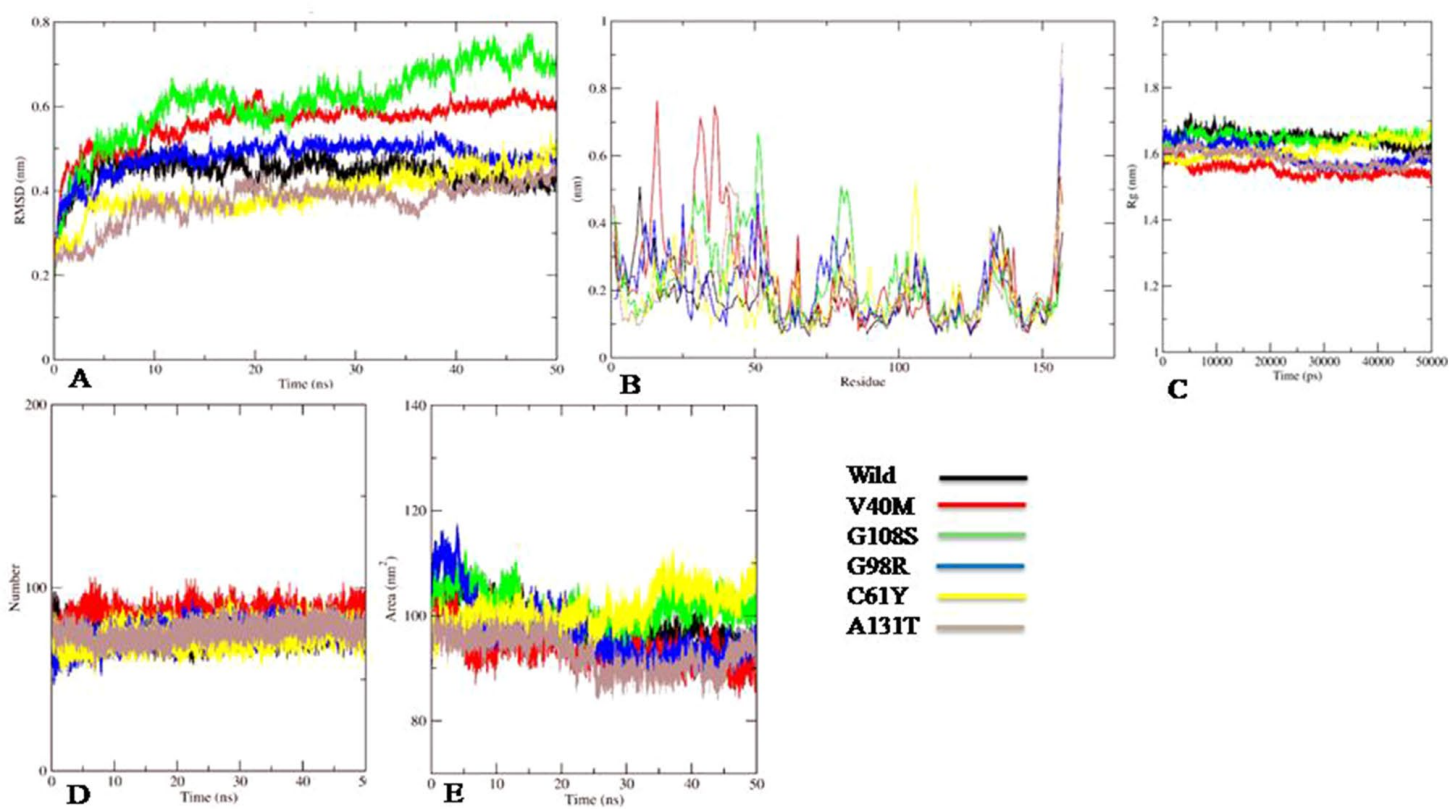
Molecular dynamic simulation of wild (black) and mutant variants V40M **(A)** RMSD analysis, **(B)** RMSF analysis, **(C)** radius of gyration analysis, **(D)** hydrogen bond analysis, **(E)** SASA analysis.

MD simulation based on stability, flexibility, compactness and hydrogen bonds monitored the effect of deleterious nsSNPs on wild type YPEL3 and its variants (Fig. 2). The average values of RMSD, RMSF, Radius of gyration, h-bond and SASA are represented in Table S12.

The RMSD analysis of backbone residues analysed the deviation of mutant structures from wild variant based on the convergence of simulation trajectories (Figure S6). All the trajectories revealed significant variation with the highest observed variation in the mutant V40M and G108S with an average RMSD value of 0.56 and 0.61 nm. The RMSD value for wild type ranged from 0.0004 to 0.530 nm with an average value of 0.44 nm. After a spike of approximately 0.45 nm till 2.5 ns, steady fluctuation was observed till 50 ns in the wild type structure; however the plot showed its own characteristic fluctuations at each time interval. For the mutant V40M, significant deviation was observed for the period of first 2 ns followed by a steady plot till 9th ns. Since then, the trajectory showed a subsequent rise in deviation from the wild structure. The mutant C61Y and A131T showed a lower value of RMSD trajectory when compared to the wild variant for the first 35 to 40 ns after which C61Y showed an increase whereas A131T followed the similar trajectory to the wild variant. The mutant G108S followed a similar trajectory for about first 3.6 ns after which a sharp rise and deviation was observed with wild variant till the completion of simulation i.e. 50 ns. The trajectory of mutant G98R showed a similar plot with respect to the wild variant for approximately first 19 ns and thereafter minor fluctuations were observed. Concluding the trajectory analysis, the mutants V40M and G108S imparted in decreasing the stability of protein along with mutant G98R, whereas the mutant C61Y and A131T facilitated an increased stability of protein.

RMSF analysis depicted a fluctuating trajectory for the wild variant with an average RMSF value of 0.17 nm. In case of mutation V40M, the RMSF value of mutant showed significant increase in the peak from residue 14–75 and rest most of the residues followed the similar pattern as the wild variant except the residue 156 with a higher RMSF value of 0.54 nm. Subsequently, the observed RMSF value for mutation G108S showed higher peaks from residue 25–53 and 73–103 with the wild variant. In case of V40M and G108S, maximum number of residues of mutant showed higher RMSF value in compare to the wild variant revealing increase in flexibility of the protein with an average RMSF of 0.24 nm and 0.22 nm. Further the mutants G98R, C61Y showed differential pattern of RMSF trajectory in compare to wild variant with an average RMSF of 0.21 nm and 0.19 nm contributing to the increased flexibility of the protein. The fluctuation in mutant A131T were in accordance to the wild variant for the majority of residues except for residue 154 to 157 with exceptionally high peak. The RMSF analysis resulted to demonstrate that mutants V40M, G108S and G98R showed significant rise in average value, whereas mutants C61Y and A131T did not show a major change in compare to the wild protein. All these observations suggested that the mutations alter the conformation and change the flexibility of protein (Figure S7). The Rg value of wild protein ranged between 1.490 and 1.605 nm with an average value of 1.64 nm. However, the Rg value of mutants V40M, G98R, C61Y and A131T were lower than the values of wild protein. The mutant G108S showed similar trajectory as of wild with slight increase in values at the end of simulation with an average Rg value of 1.65 nm. The gyration analysis for all the mutants revealed that the mutant V40M showed higher level of compaction with an average value of 1.55 nm followed by G98R, C61Y and A131T in compare to the wild protein, whereas the mutants G108S had approximately similar compactness as of wild protein (Figure S8).

The h-bond analysis revealed no significant differences in the number of intermolecular hydrogen bonds over the period of 50 ns between the wild and mutants except in case of V40M. For the first 15 ns, a slight decrease in number of h-bonds was observed in mutants G108S, G98R and C61Y. Finally, in mutation V40M, a significant increase in rigidity of protein was observed and so the change in geometry of protein, while other mutations did not impart any such change (Figure S9).

The SASA analysis showed average estimates 93.37 nm^2^, 96.94 nm^2^ and 92.73 nm^2^ in case of mutants V40M, G98R and A131T, respectively in compare to the wild variant with average estimates of 98.39 nm^2^. In case of mutant G108S, at the start and end of simulation spike in trajectory was observed with an average value of 101.44 nm^2^, whereas in case of G98R overall trajectory was lower than the wild except at the start of simulation. Exceptionally, C61Y showed differential pattern where starting with the lower values, the trajectory significantly elevated higher over the period of time than the wild variant. This indicated that the mutation V40M, G98R and A131T reduced the overall accessible surface area of protein while the mutation G108S and C61Y increased the available surface area for the solvents (Figure S10).

### Kaplan–Meier plotter analysis

**Figure 3:**
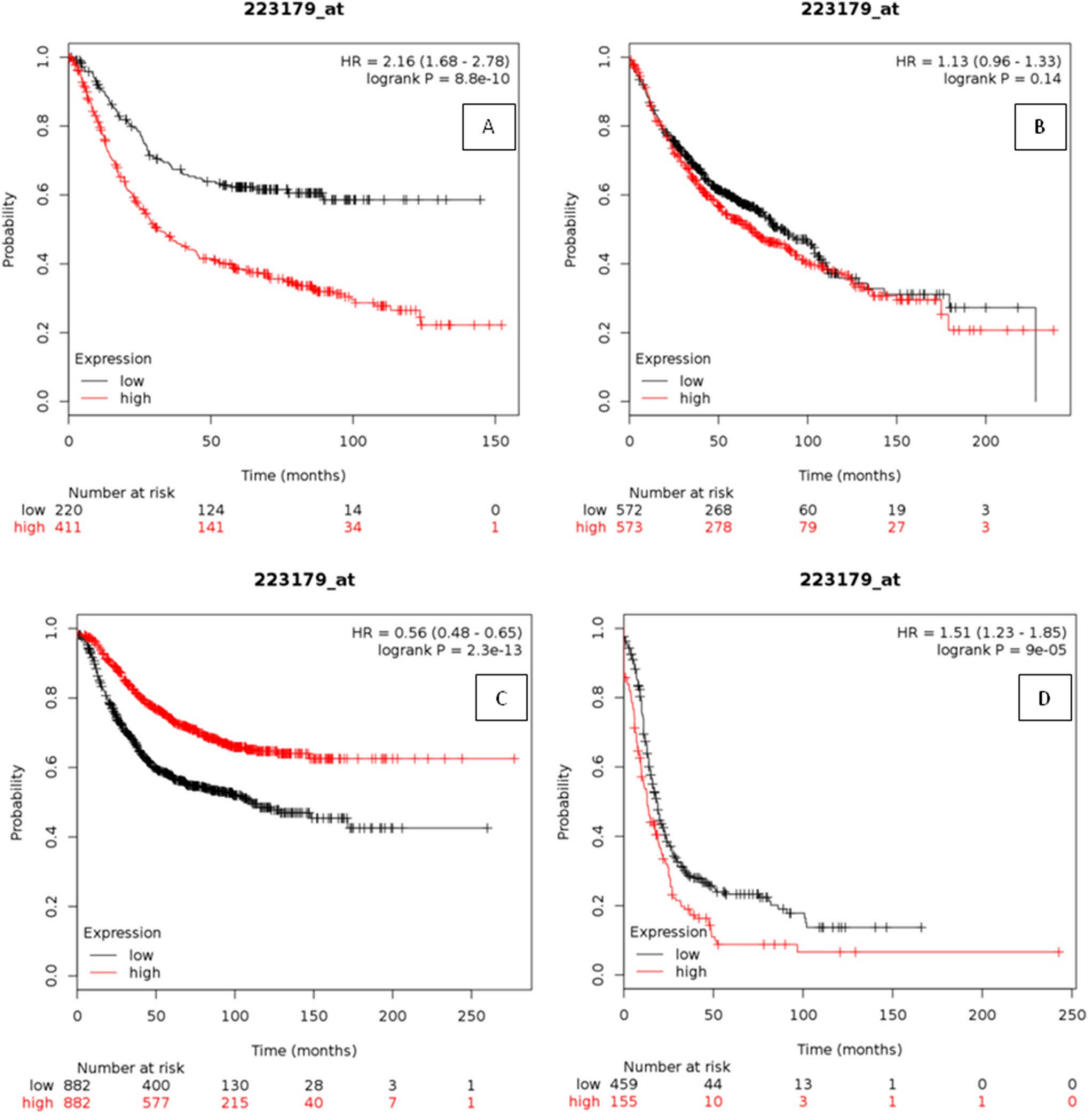
Survival rate based on the microarray gene expression. **(A)** Correlation graph between deregulation of YPEL3 gene and gastric cancer; **(B)** correlation graph between deregulation of YPEL3 gene and lung cancer; **(C)** correlation graph between deregulation of YPEL3 gene and breast cancer and **(D)** correlation graph between deregulation of YPEL3 gene and ovarian cancer.

The Kaplan–Meier plotter analysis showed high expression of the YPEL3 gene and was positively correlated with the more number of patients at high risk in Gastric cancer patients (Fig. 3A). Whereas, in Lung and Breast cancer, no significant correlation was obtained between the expression level of YPEL3 gene and the survival rate of patients (Fig. 3B, C). However, in Ovarian cancer patients, a significant decrease in the number of risks (more survival rate) with high expression of YPEL3 gene was observed (Fig. 3D).

## Discussion

In recent years, public databases like dbSNP, HGMD, and HGVbase have been enriched with the SNP data corresponding to different genes. These database contain information regarding various aspect of genetic disorders and on intensive investigations, one can also extract brief knowledge about mutations in the genetic markers and their various effects in human. There are a few studies that reported the altered form of genetic markers, may lead to different diseases^3–7^. This demands an extensive study of all the genes and their correlation with genetic disorders. In this regard, we found that YPEL3 is one of the least explored genetic markers in the context of investigating effects of nsSNPs to human disease. However, a few earlier studies have reported varying effects of nsSNPs in several genes in human^3–7,12–21^. As protein function is directly related to the tertiary structure of the protein, so any variation of amino acid in the structure could alter the physiological effect. Consequently, alteration in the physiology of protein may affect its growth-suppressive properties. Investigation of these polymorphisms at the protein level and its potential biological effect is necessary as YPEL3 down regulation in various tumor conditions could prove it to be an important molecular target for anti-cancer therapies.

In this study, we predicted 10 deleterious nsSNPs in the YPEL3 gene and their effect on protein properties. All variants were predicted to decrease protein stability by I-Mutant and MuPro servers except in the case of variant R57L (MuPro). In the selected 10 nsSNPs, the ConSurf server predicted 9 highly conserved nsSNPs with a conservation score range from 7 to 9. Further, the substituted positions V40, C61, G98, G108, D114, A131, and I145 were buried with structural significance in protein structure while positions R57, E129 were exposed with E129 having functional significance. Although the mutation V40 showed a conservation score of 2, but this position was taken into account for further analysis since the calculation was performed on less than 6 non gaped homologous sequences. Out of these 10 substitution sites, C61 was the metal-binding site for zinc motifs which was highly conserved site and any change at C61 could have a pathogenic effect and alter the protein function. These variants were further evaluated for PTM analysis.

Of all the PTM sites present on protein sequence, none of them was common with the nsSNPs location except R57L for methylation. But since the R57L did not show any significant result with respect to the stability of the protein, therefore this PTM site was not considered damaging to the native protein. Further, all 3D structures of YPEL3 protein and its mutants except for R57L, D114N, A131V, and E129G showed a greater degree of variation with native protein structure which signifies structural changes. But among the selected variants, mutant I145T showed high variation with low conservation score in compare to other structures, thus only five structures i.e. V40M, G108S, G98R, C61Y and A131T were explored further. The mutations G98R and G108S replaced Glycine which is one of the most flexible amino acids disturbing the required rigidity of protein. The size difference at position 61 between Cysteine and Tyrosine can disturb the binding of Zinc metal ion. Based on the change in the rigidity, charge and size differences, all selected mutations were predicted damaging to the protein. Secondary structure analysis revealed an absence of Pi-helix in the mutant variants to affect the protein folding and stability. To assess the mutational effect of YPEL3 protein on its biological functional partners, gene–gene interaction analysis revealed that gene WDR26 was the highly interacting gene and DOK7 was the physically interacting gene, and therefore any functional or structural change in YPEL3 could affect the interaction between them. MD Simulation analysis assessed the native behavior of protein in the simulated environment on the basis of stability, flexibility and dimensions of protein as a function of time. The RMSD and RMSF analysis in agreement to each other revealed that the variants V40M, G108S and G98R decreased the stability and increased the flexibility of protein, whereas variants C61Y and A131T showed increase in stability and decrease in the flexibility of protein. The radius of gyration and hydrogen bond analysis revealed that the variant V40M showed higher level of compactness throughout the time, whereas the other variants showed differential level of compactness. The SASA analysis revealed that all the variants changes the native conformation of protein and hence possibly responsible for change in function of protein. Based on the simulation study, we demonstrated that the variants V40M, G108S, G98R, C61Y and A131T imparted changes in the native conformation or structure of the YPEL3 protein in any sense of behavior and hence predicted to affect the protein function and structure in deleterious manner. Kaplan–Meier plotter analysis revealed that the deregulation of YPEL3 gene had antagonistic effects on the survival rate of gastric and ovarian cancer patients. This correlation of YPEL3 deregulation with gastric and ovarian cancer may have an indirect relation with the predicted damaging mutations in YPEL3 gene. The YPEL3 gene was thus found to be an important prognostic marker in case of Gastric and Ovarian cancer patients. It is evident from the results that YPEL3 gene expression was not linked with the gender of the cancer patients as both Breast and Ovarian cancers are gender-specific but only Ovarian cancer patients showed a significant correlation with YPEL3 gene deregulation. However, the expression of YPEL3 gene is regulated by several other genes also^11^.

The findings of this study will provide clarity in understanding the mutational effect of deleterious nsSNPs on YPEL3 protein and to elucidate their role in different associated diseases. In this study, we utilized highly reliable and widely used computational tools with molecular dynamic simulation analysis and determined five deleterious mutations with both structural and functional consequences on native YPEL3 protein. The results have the potential to pave new insights in drug target identification and Biomarker assessment. However, in-vitro functional assessments are required to ascertain the effects of amino acid change in the native protein. Besides the functional changes, assessment of structural changes due to mutations are also required and therefore 3D crystal structures of the protein is a prerequisite for future studies. Since, the present study investigated the nsSNPs effects on YPEL3 protein, was solely based on the predictions using computational approaches and therefore, experimental validation and comprehensive clinical studies with the inclusion of real time case histories is required for further evaluation and exploring aid to cancer therapies.

## Materials and methods

### Data mining

The nucleotide sequence of the Human YPEL3 gene was retrieved from the GenBank (https://www.ncbi.nlm.nih.gov/gene/83719). The amino acid sequence of protein YPEL3 isoform 2 composed of 157 AA residues was obtained from the Uniprot database (Uniprot ID: P61236). All SNPs along with their metadata like position, residue change and global minor allele frequencies (MAF) were retrieved from NCBI dbSNP^22^ and then the nsSNPs were filtered out from the function class of database (Accessed on 29th Oct, 2019).

### Identification of the most deleterious nsSNPs

We used two bioinformatics tools, i.e. PredictSNP2^23^ and PredictSNP1^24^ to envisage the deleterious effect of nsSNPs on protein function. These tools are consensus classifier that enabled us to access and predict through six integrated tools in (CADD, DANN, FATHMM-MKL, FunSeq2, GWAVA, and PredictSNP2) in PredictSNP2 and nine prediction tools (MAPP, PhD SNP, PolyPhen-1, Polyphen-2, SIFT, SNAP, Panther, nsSNPanalyzer and PredictSNP1) in PredictSNP1. The prediction results of predictSNP2 were exported to PredictSNP1 along with both neutral and deleterious nsSNPs.

### Assessment of protein stability

To predict the stability of protein, we used two computational tools i.e. I Mutant 2.0^25^ and MuPro^26^. I Mutant uses a support vector machine-based web server for prediction of protein stability based on any single site mutation. This tool uses data from ProTherm^27^ that is the most comprehensive database of protein mutations derived from experimental data. I-Mutant predicts the change in stability by calculating the change in DDG. It further defines whether these changes increase or decrease the stability of the protein. Negative DDG indicates that protein stability decreases and positive DDG indicates that protein stability increases. Based on the signature of stability change, it gives the Reliability index value (RI value) ranging from 0 to 10, where 10 depict the higher reliability. Accordingly, MuPro predicts the effect of a single-site mutation on the stability of protein, based on a set of machine learning programs. The results are based on two machine learning methods, viz., Support vector machine and neural networks. MuPro predicts the effect of mutation on protein stability on the basis of the value of energy change (ΔΔG). It also predicts the sign of energy change using neural networks and support vector machines. This tool is capable of predicting protein stability without the availability of tertiary structure. We submitted protein YPEL3 sequence to these tools to predict the effect of deleterious nsSNPs on the protein structure providing temperature conditions of 25 ºC and ph 7.0.

### Prediction of conserved residues

To predict the evolutionary conservation of the amino acids in protein sequence, we used ConSurf bioinformatics tool^28^ that provide evolutionary profiles of each of the amino acids in the protein, based on phylogenetic relations between homologous sequences. The tool also predicts the conservation score for each amino acid residue ranging from 1–9, where the score 1–3 denotes variable residues, 4–6 denotes medium conserved and scores 7–9 depict highly conserved residue. These scores indicate the degree to which the amino acids are evolutionary conserved. For this analysis, we first submitted the Uniprot protein sequence of YPEL3 protein in BLASTp against the UniProt database in NCBI and selected those sequences having an identity of more than 50% for further analysis. After the multiple sequence alignment of these sequences, the MSA file was then submitted to ConSurf along with the protein FASTA sequence.

### Prediction of PTM sites

A post translational modification is crucial for cell signalling and affects the function of the protein. Based on the previous studies^29^, Methylation and Ubiquitylation PTM affects the functioning and regulation of the YPEL3 gene. The methylation sites in Human Yippee like 3 protein sequence were predicted using GPS-MSP v.1^30^ and PMeS^31^ tools. GPS-MSP v.1 predicts mono, symmetry di-, asymmetry di-methylation types specific for arginine residues and mono, di and trimethylation type-specific to lysine residues. It is commonly used in the prediction of potential methylation sites with a threshold value of 0.5. Whereas, PMeS tool identifies methylation sites based on the enhanced feature encoding scheme and supports vector machine. Similarly, the phosphorylation sites at tyrosine, threonine and serine residues were predicted using GPS 5.0^32^ and NetPhos 3.1^33^. Higher value in GPS 5.0 predicts higher chances of residues to get phosphorylated, whereas NetPhos 3.1 uses ensemble approach of several neural networks to predict residue scores with a threshold of 0.5. BDM-PUB^34^ and UbPred^35^ are common tools for the prediction of protein Ubiquitylation sites. A balanced cut-off performance selection was used in BDM-PUB, based on Bayesian Discriminant method to predict Ubiquitylation positions. UbPred considers a score of ≥ 0.62 to predict Ubiquitylated residues.

### Structure modeling of wild and mutant protein

The 3D models of wild and its mutants with most deleterious nsSNPs of YPEL3 protein were generated using Phyre2^36^. The template selected by Phyre2 to predict the 3D models of both native and mutant proteins was c4v30A. The 3D structure of native and mutant proteins were then compared using the TM-Score web tool. The TM- Score is a popular web tool that provides Template modeling scores (TM-score) and Root mean square deviation (RMSD) value on comparison of native and mutant variant protein^37,38^. TM-score provides a range of 0–1 where 0 signifies lower structural similarity and 1 signifies higher structural similarity. Likewise higher RMSD value indicates greater variation between native and mutant protein structures^39^. The mutants having higher RMSD values were submitted to I-Tasser^40^, which is a very advanced tool for protein structure prediction and analysis. The predicted structures by I-Tasser were verified using ERRAT programme^41^ which is extensively used in verifying protein structures. Finally, the native structure and selected mutant structures of YPEL3 protein were visualized and superimposed in Chimera 1.10.1^42^.

### Validation of modelled proteins

The native and mutant modelled structures were verified using ProSA^43,44^ and ProCheck tool^45^. The ProSA tool predicts the overall quality of modelled structure on the basis of Z-Score. If the Z-score of the models lies outside the scores of proteins with similar size, the chance of error increases in the predicted structure. Likewise, ProCheck tool assess the overall quality of model by identifying the percentage of residues in most favored regions, additional allowed regions, generously allowed regions and disallowed regions. Based on these results, the models with Z-score in the range of proteins of similar size and having the disallowed region percentage less than 10% were selected for further analysis.

### Structural insight of protein

The structural change and its effect on protein structure due to variants were predicted by a mutant analysis server i.e. Project HOPE^46^. It explained the structural effect of variants on native protein using UniProt and Das prediction servers.

### Position level analysis of secondary structure

To assess the position level variation in secondary structure of wild and mutant proteins, STRIDE programme was used^47^. It utilizes the atomic coordinates of the modelled structure to assign the secondary structure codes, which is one of the most complex processes in compare to other programmes.

### YPEL3 functional interaction

Interaction and association of YPEL3 gene with other genes was studied using STRING v.11.0^48^ and Gene MANIA database^49^. String predicts gene interaction on the basis of neighborhood, gene fusion, concurrence, co expression, experiments, database, text mining and homology. Based on these parameters, scores are assigned from 0 to 1 to each functional partner genes where 0 denotes lower interaction and 1 denotes higher interaction. Gene MANIA predicts association network and functional interaction based on co-expression, co-localization, pathway, genetic interaction, physical interaction and similarity of protein domains.

### Molecular dynamic (MD) simulation

Molecular dynamic simulation was performed using GROMACS 2020.1^50^-Ubuntu-2020.1-1 version and Linux 4.4.0 package on Intel Core i7 processor, 32 GB RAM system. Structures of wild and mutant proteins were used as a starting structure of MD simulation and the solvation of cubic systems were done at 10 Å radius with point charge SPCE water molecules. Since the structures were found to be positively charged at the physiological pH, three chloride (Cl^-^) ions were added using “genion” tool of GROMACS to the simulation system in order to neutralise the charge of the systems. These solvated neutral systems were subjected for energy minimization for 500,00 steps using steepest descent algorithm of OPLS-AA/L all-atom force field to relax the structure and ensure absence of steric clashes. Later to control the temperature and compute the electrostatic interaction, Berendsen temperature coupling^51^ and Particle Mesh Ewald method^52^ was used. The compressibility range to maintain the pressure at 1 atm was 4.5e − 5 atm. LINCS algorithm was used for constraining the bond lengths for a time step of 2 fs. Finally, MD simulation was performed for 50 ns. Thereafter, a comparative analysis was performed between the wild and mutant structures based on Root mean square deviation (RMSD), Root mean square fluctuation (RMSF), Radius of gyration (Rg), Hydrogen bond and Solvent accessible surface area (SASA) analysis by using gmx rms, gmx rmsf , gmx gyrate, gmx hbond and gmx sasa. All these analysis were represented in the form of plots using XMGRACE program^53^.

### Kaplan–Meier plotter analysis

Previous studies^11^ have mentioned that YPEL3 is under expressed in several types of cancer. So the assessment of the YPEL3 gene on the survival of several cancer types is crucial to Human medical genetics. Kaplan Meier plotter analysis^54^ is capable to assess the effect of 540,00 genes in the survival of 21 types of cancer using the data of 133,16 patients (6,234-Breast, 2,190-Ovarian, 3,542-Lung and 1,440-Gastric). The databases used by Kaplan Meier plotter were Gene Expression Omnibus (GEO), European Genome-phenome Archive (EGA) and The Cancer Genome Atlas (TCGA). In this analysis, the probe used for the YPEL3 gene was 223179_at. In this study, the overall analysis was done on 1,764 Breast, 614 Ovarian, 1,926 Lung and 876 Gastric cancer patients. Two groups of patients (High and Low expression level) based on median value for each cancer types and based on this, survival was assessed. A quality control standard was maintained by excluding the biased arrays and a p-value below 0.5 was considered significant.

## Supplementary information

Supplementary Information.

## Data Availability

All relevant data is included in the manuscript.
