# Supplementary material for: Exploring the effect of nsSNPs in human YPEL3 gene in cellular senescence
Source: Sci Rep. 2020 Sep 17;10:15301. doi: 10.1038/s41598-020-72333-8 (PMC7498449; doi:10.1038/s41598-020-72333-8)
Supplement: Supplementary file 1 — Supplementary Information. [file 41598_2020_72333_MOESM1_ESM.docx]

| **Table S1. PredictSNP2 result of 75 SNPs in YPEL3 gene.**  Running title: Exploring the effect of nsSNP in Human YPEL3 gene in cellular senescence.  Authors: Abhishek Singh^1^*, Mukesh Thakur^1^*, Sujeet Kumar Singh^1^ , Lalit Kumar Sharma^1^ and Kailash Chandra^1^ | | | | | | | | | | | | | | |
| --- | --- | --- | --- | --- | --- | --- | --- | --- | --- | --- | --- | --- | --- | --- |
| **Variant** | **SNP ID** | **Exonic. func** | **PredictSNP2** | | **CADD** | | **DANN** | | **FATHMM** | | **FunSeq2** | | **GWAVA** | |
|  |  |  | **Prediction** | **Score** | **Prediction** | **Score** | **Prediction** | **Score** | **Prediction** | **Score** | **Prediction** | **Score** | **Prediction** | **Score** |
| 16 : 30095395 | rs184524218 | nonsynonymous | neutral | -1 | deleterious | 22.3 | neutral | 0.9795 | neutral | 0.7547 | neutral | 2 | deleterious | 0.57 |
| 16 : 30092741 | rs200869087 | nonsynonymous | neutral | 0.0101 | deleterious | 22.7 | neutral | 0.9847 | deleterious | 0.8976 | neutral | 2 | deleterious | 0.64 |
| 16 : 30095376 | rs201763714 | nonsynonymous | deleterious | 0.0973 | deleterious | 22.3 | deleterious | 0.9981 | neutra | 0.8098 | neutral | 2 | deleterious | 0.62 |
| 16 : 30092736 | rs202058576 | nonsynonymous | neutral | -0.0225 | deleterious | 23 | neutral | 0.9097 | deleterious | 0.9859 | neutral | 2 | deleterious | 0.64 |
| 16 : 30095416 | rs368612155 | nonsynonymous | neutral | -0.0026 | deleterious | 21.7 | neutral | 0.9812 | deleterious | 0.9155 | neutral | 2 | deleterious | 0.58 |
| 16 : 30094836 | rs370953161 | nonsynonymous | neutral | 0.0338 | deleterious | 22.6 | neutral | 0.9892 | deleterious | 0.9073 | neutral | 2 | deleterious | 0.54 |
| 16 : 30092792 | rs373399618 | nonsynonymous | deleterious | 1 | deleterious | 22.9 | deleterious | 0.9991 | deleterious | 0.9875 | neutral | 2 | deleterious | 0.66 |
| 16 : 30095313 | rs374762947 | nonsynonymous | deleterious | 1 | deleterious | 22.6 | deleterious | 0.9958 | deleterious | 0.904 | neutral | 2 | deleterious | 0.54 |
| 16 : 30092782 | rs541341176 | synonymous | deleterious | 1 | deleterious | 22.5 | deleterious | 0.9964 | deleterious | 0.9446 | deleterious | 2 | deleterious | 0.67 |
| 16 : 30095266 | rs568854299 | nonsynonymous | deleterious | 1 | deleterious | 22.4 | deleterious | 0.9969 | deleterious | 0.956 | neutral | 2 | ? | 0.45 |
| 16 : 30095440 | rs746153617 | nonsynonymous | neutral | -1 | neutral | 11.39 | neutral | 0.9215 | neutral | 0.0273 | neutral | 1 | ? | 0.48 |
| 16 : 30095370, | rs747250995 | nonsynonymous | neutral | -0.0795 | deleterious | 22 | deleterious | 0.996 | neutral | 0.5628 | neutral | 2 | deleterious | 0.62 |
| 16 : 30095340, | rs749362986 | nonsynonymous | neutral | 0.0064 | deleterious | 22.4 | deleterious | 0.998 | neutral | 0.6569 | neutral | 2 | ? | 0.5 |
| 16 : 30095268, | rs751446326 | nonsynonymous | neutral | 0.0679 | deleterious | 21.8 | deleterious | 0.9976 | neutral | 0.8065 | neutral | 2 | ? | 0.43 |
| 16 : 30092720 | rs752040143 | nonsynonymous | deleterious | 1 | deleterious | 22.8 | deleterious | 0.9965 | deleterious | 0.9877 | neutral | 2 | deleterious | 0.6 |
| 16 : 30095301 | rs753385457 | nonsynonymous | deleterious | 1 | deleterious | 22.5 | deleterious | 0.9932 | deleterious | 0.9306 | neutral | 2 | ? | 0.53 |
| 16 : 30094866 | rs753898977 | nonsynonymous | deleterious | 0.1072 | deleterious | 22.1 | deleterious | 0.9992 | neutral | 0.7577 | neutral | 2 | deleterious | 0.59 |
| 16 : 30095289 | rs755667144 | nonsynonymous | neutral | 0.0563 | deleterious | 22.5 | deleterious | 0.9968 | neutral | 0.884 | neutral | 2 | deleterious | 0.54 |
| 16 : 30095419 | rs756625481 | nonsynonymous | neutral | -1 | deleterious | 22.2 | neutral | 0.97 | neutral | 0.4067 | neutral | 2 | deleterious | 0.58 |
| 16 : 30092750 | rs757003466 | nonsynonymous | deleterious | 1 | deleterious | 22.7 | deleterious | 0.998 | deleterious | 0.9888 | neutral | 2 | deleterious | 0.62 |
| 16 : 30095407 | rs758031738 | nonsynonymous | deleterious | 0.0973 | deleterious | 22.3 | deleterious | 0.9982 | neutral | 0.8457 | neutral | 2 | deleterious | 0.55 |
| 16 : 30095272 | rs759352096 | nonsynonymous | neutral | -0.0608 | deleterious | 21.6 | neutral | 0.9672 | deleterious | 0.9092 | neutral | 2 | ? | 0.48 |
| 16 : 30095365 | rs759413482 | nonsynonymous | deleterious | 1 | deleterious | 22.6 | deleterious | 0.9987 | deleterious | 0.9138 | neutral | 2 | ? | 0.53 |
| 16 : 30094851 | rs760745635 | nonsynonymous | deleterious | 1 | deleterious | 22.6 | deleterious | 0.9986 | deleterious | 0.9116 | neutral | 2 | ? | 0.53 |
| 16 : 30095379 | rs764063495 | nonsynonymous | neutral | -1 | deleterious | 20.5 | neutral | 0.9465 | neutral | 0.1915 | neutral | 1 | deleterious | 0.57 |
| 16 : 30094867 | rs765168951 | nonsynonymous | neutral | -1 | deleterious | 21.4 | neutral | 0.9557 | neutral | 0.7915 | neutral | 2 | deleterious | 0.56 |
| 16 : 30092715 | rs767025068 | nonsynonymous | deleterious | 1 | deleterious | 22.9 | deleterious | 0.9983 | deleterious | 0.9849 | neutral | 2 | deleterious | 0.58 |
| 16 : 30095374 | rs767366278 | nonsynonymous | neutral | -1 | deleterious | 22.1 | neutral | 0.987 | neutral | 0.5515 | neutral | 2 | deleterious | 0.63 |
| 16 : 30095353 | rs771297278 | nonsynonymous | neutral | -0.0477 | deleterious | 22.3 | deleterious | 0.9953 | neutral | 0.6697 | neutral | 2 | ? | 0.51 |
| 16 : 30095442 | rs772422350 | nonsynonymous | neutral | -1 | neutral | 13.68 | neutral | 0.9895 | neutral | 0.5541 | neutral | 1 | ? | 0.48 |
| 16 : 30095476 | rs774080188 | nonsynonymous | neutral | -1 | neutral | 15.13 | neutral | 0.8272 | neutral | 0.2477 | neutral | 1 | deleterious | 0.25 |
| 16 : 30095439 | rs774730954 | nonsynonymous | neutral | -1 | neutral | 15.11 | neutral | 0.9807 | neutral | 0.1665 | neutral | 1 | ? | 0.45 |
| 16 : 30095446 | rs775191908 | nonsynonymous | neutral | -1 | neutral | 13.27 | neutral | 0.96 | neutral | 0.5326 | neutral | 1 | ? | 0.51 |
| 16 : 30095475 | rs776592208 | nonsynonymous | neutral | -1 | neutral | 16.27 | neutral | 0.977 | neutral | 0.8246 | neutral | 2 | deleterious | 0.32 |
| 16 : 30092748 | rs777945028 | Stopgain | deleterious | 0.622 | deleterious | 22.8 | deleterious | 0.9963 | deleterious | 0.9868 | neutral | 3 | deleterious | 0.6 |
| 16 : 30092735 | rs781393670 | nonsynonymous | neutral | -0.0019 | deleterious | 23.1 | neutral | 0.9414 | deleterious | 0.989 | neutral | 2 | deleterious | 0.61 |
| 16 : 30095314 | rs781672800 | nonsynonymous | deleterious | 0.145 | deleterious | 22.4 | deleterious | 0.9992 | neutral | 0.8263 | neutral | 2 | deleterious | 0.56 |
| 16 : 30094833 | rs936261369 | nonsynonymous | deleterious | 1 | deleterious | 22.5 | deleterious | 0.9992 | deleterious | 0.9123 | neutral | 2 | deleterious | 0.55 |
| 16 : 30095445 | rs104704071 | nonsynonymous | neutral | -1 | neutral | 13.74 | neutral | 0.9851 | neutral | 0.5116 | neutral | 1 | ? | 0.51 |
| 16 : 30095383 | rs115670638 | nonsynonymous | neutral | 0.0607 | deleterious | 22.2 | deleterious | 0.997 | neutral | 0.8239 | neutral | 2 | deleterious | 0.62 |
| 16 : 30095298 | rs115985789 | nonsynonymous | neutral | -1 | deleterious | 22.2 | neutral | 0.9536 | neutral | 0.6176 | neutral | 2 | deleterious | 0.59 |
| 16 : 30095449 | rs117157782 | nonsynonymous | neutral | -1 | neutral | 13.89 | neutral | 0.981 | neutral | 0.5123 | neutral | 1 | ? | 0.5 |
| 16 : 30095443 | rs117530534 | nonsynonymous | neutral | -1 | neutral | 11.78 | neutral | 0.9247 | neutral | 0.6605 | neutral | 1 | ? | 0.5 |
| 16 : 30095373 | rs120029665 | nonsynonymous | neutral | -0.0777 | deleterious | 22.3 | deleterious | 0.9957 | neutral | 0.6028 | neutral | 2 | deleterious | 0.63 |
| 16 : 30092783 | rs121794506 | nonsynonymous | neutral | -0.0026 | deleterious | 22.6 | neutral | 0.983 | deleterious | 0.9301 | neutral | 2 | deleterious | 0.62 |
| 16 : 30094881 | rs123481349 | nonsynonymous | deleterious | 1 | deleterious | 22.5 | deleterious | 0.9988 | deleterious | 0.9115 | neutral | 2 | deleterious | 0.59 |
| 16 : 30095404 | rs123807045 | nonsynonymous | neutral | 0.0391 | deleterious | 22.2 | deleterious | 0.9976 | neutral | 0.7598 | neutral | 2 | deleterious | 0.54 |
| 16 : 30094872 | rs128096618 | nonsynonymous | deleterious | 0.145 | deleterious | 22.5 | deleterious | 0.9991 | neutral | 0.8861 | neutral | 2 | deleterious | 0.57 |
| 16 : 30095362 | rs128309247 | nonsynonymous | neutral | 0.0515 | deleterious | 22.3 | deleterious | 0.9985 | neutral | 0.7161 | neutral | 2 | ? | 0.53 |
| 16 : 30092793 | rs129554256 | nonsynonymous | deleterious | 1 | deleterious | 22.9 | deleterious | 0.9989 | deleterious | 0.9817 | neutral | 2 | deleterious | 0.61 |
| 16 : 30095269 | rs129559908 | nonsynonymous | neutral | 0.0607 | deleterious | 22.6 | deleterious | 0.997 | neutral | 0.848 | neutral | 2 | ? | 0.5 |
| 16 : 30095482 | rs129985775 | nonsynonymous | neutral | -1 | neutral | 7.564 | neutral | 0.6031 | neutral | 0.0379 | neutral | 1 | deleterious | 0.31 |
| 16 : 30095413 | rs131110667 | nonsynonymous | neutral | -1 | deleterious | 21.5 | neutral | 0.9816 | neutral | 0.8468 | neutral | 2 | deleterious | 0.62 |
| 16 : 30092799 | rs131637644 | nonsynonymous | deleterious | 1 | deleterious | 22.6 | deleterious | 0.9982 | deleterious | 0.9854 | neutral | 2 | deleterious | 0.59 |
| 16 : 30095133 | rs133020448 | nonsynonymous | deleterious | 1 | deleterious | 22.5 | deleterious | 0.9935 | deleterious | 0.9432 | neutral | 2 | deleterious | 0.58 |
| 16 : 30095256 | rs135426813 | nonsynonymous | deleterious | 1 | deleterious | 22 | deleterious | 0.9972 | deleterious | 0.936 | neutral | 2 | ? | 0.52 |
| 16 : 30095403 | rs137617040 | nonsynonymous | deleterious | 0.0737 | deleterious | 22.1 | deleterious | 0.9982 | neutral | 0.777 | neutral | 2 | deleterious | 0.63 |
| 16 : 30095386 | rs137726377 | nonsynonymous | neutral | -1 | deleterious | 21.5 | neutral | 0.9028 | neutral | 0.769 | neutral | 2 | deleterious | 0.6 |
| 16 : 30095295 | rs137895313 | nonsynonymous | neutral | -1 | deleterious | 22.3 | neutral | 0.9701 | neutral | 0.8835 | neutral | 2 | ? | 0.53 |
| 16 : 30095477 | rs138665653 | nonsynonymous | neutral | -1 | neutral | 15.59 | neutral | 0.977 | neutral | 0.7312 | neutral | 2 | deleterious | 0.26 |
| 16 : 30095467 | rs138919801 | nonsynonymous | neutral | -1 | neutral | 15.61 | neutral | 0.9791 | neutral | 0.1608 | neutral | 2 | ? | 0.24 |
| 16 : 30095398 | rs139331488 | nonsynonymous | neutral | -0.1412 | deleterious | 22.1 | deleterious | 0.9954 | neutral | 0.3432 | neutral | 2 | deleterious | 0.62 |
| 16 : 30092722 | rs140546218 | synonymous | deleterious | 0.4841 | deleterious | 22.1 | deleterious | 0.897 | deleterious | 0.8737 | neutral | 0 | deleterious | 0.55 |
| 16 : 30095116 | rs140981423 | nonsynonymous | deleterious | 1 | deleterious | 22.3 | deleterious | 0.9986 | deleterious | 0.9321 | neutral | 2 | deleterious | 0.62 |
| 16 : 30092713 | rs141889835 | nonsynonymous | neutral | -0.1243 | deleterious | 22 | neutral | 0.939 | deleterious | 0.9313 | neutral | 2 | deleterious | 0.6 |
| 16 : 30094832 | rs142226332 | nonsynonymous | deleterious | 1 | deleterious | 22.1 | deleterious | 0.9982 | deleterious | 0.9094 | neutral | 2 | deleterious | 0.56 |
| 16 : 30092726 | rs142297015 | nonsynonymous | deleterious | 1 | deleterious | 22.6 | deleterious | 0.9958 | deleterious | 0.9839 | neutral | 2 | deleterious | 0.63 |
| 16 : 30092734 | rs143027803 | nonsynonymous | deleterious | 0.1487 | deleterious | 23.3 | neutral | 0.9877 | deleterious | 0.9848 | neutral | 2 | deleterious | 0.59 |
| 16 : 30094808 | rs143717503 | nonsynonymous | neutral | 0.0354 | deleterious | 22.1 | neutral | 0.9836 | deleterious | 0.948 | neutral | 2 | deleterious | 0.61 |
| 16 : 30094829 | rs144628579 | nonsynonymous | neutral | 0.0448 | deleterious | 21.8 | neutral | 0.9909 | deleterious | 0.939 | neutral | 2 | ? | 0.53 |
| 16 : 30092721 | rs146849316 | nonsynonymous | deleterious | 1 | deleterious | 23 | deleterious | 0.9973 | deleterious | 0.9872 | neutral | 2 | deleterious | 0.58 |
| 16 : 30092763 | rs148895376 | nonsynonymous | deleterious | 1 | deleterious | 22.8 | deleterious | 0.999 | deleterious | 0.9864 | neutral | 2 | deleterious | 0.59 |
| 16 : 30095316 | rs156734606 | nonsynonymous | neutral | -1 | deleterious | 22.3 | neutral | 0.9845 | neutral | 0.8329 | neutral | 2 | deleterious | 0.54 |

| **Table S2. Conservation scores of deleterious mutational sites** | | | |
| --- | --- | --- | --- |
| **Position** | **Conservation Score** | **Buried/Exposed** | **Functional/Structural** |
| V40 | 2 | Buried | - |
| R57 | 7 | Exposed | - |
| C61 | 9 | Buried | Structural |
| G98 | 9 | Buried | Structural |
| G108 | 9 | Buried | Structural |
| D114 | 9 | Buried | Structural |
| E129 | 9 | Exposed | Functional |
| A131 | 9 | Buried | Structural |
| I145 | 6 | Buried | - |

| **Table S3. Predicted methylation sites by PMeS and GPS-MSP** | | | | | |
| --- | --- | --- | --- | --- | --- |
| **PMeS** | | | **GPS-MSP** | | |
| **Position** | **Flanking residues** | **SVM Probability** | **Position** | **Flanking residues** | **Score** |
| 29 | CSPWAAP-R-VGPLPPA | 0.728545 | - | - | - |
| 41 | PPAPAMV-R-ISKPKTF | 0.506257 | - | - | - |
| 57 | AYLDDCH-R-RYSCAHC | 0.500000 | - | - | - |
| 65 | RYSCAHC-R-AHLANHD | 0.640050 | - | - | - |
| 103 | GCGPAEE-R-VLLTGLH | 0.520658 | - | - | - |

| **Table S4. Predicted Phosphorylation positions in YPEL3 gene** | | | | | |
| --- | --- | --- | --- | --- | --- |
| **NetPhos 3.1** | | | **GPS 5.0** | | |
| Serine(S) | Threonine(T) | Tyrosine(Y) | Serine(S) | Threonine(T) | Tyrosine(Y) |
| 20* | 47* | 138* | 20* | 8 | 51 |
| 23* | 107* | 143* | 23* | 47* | 87 |
| 43* |  |  | 43* | 107* | 128 |
| 60* |  |  | 60* | 122 | 138* |
| 82* |  |  | 76 | 123 | 143* |
| 134* |  |  | 78 |  |  |
| 135* |  |  | 82* |  |  |
|  |  |  | 91 |  |  |
|  |  |  | 134* |  |  |
|  |  |  | 135* |  |  |

Where * denotes phosphorylation position predicted by both NetPhos 3.1 and GPS 3.0

| **Table S5. Predicted ubiquitylation site by BDM-PUB and UbPred** | | | | | |
| --- | --- | --- | --- | --- | --- |
| **BDM-PUB** | | | **UbPred** | | |
| Position | Score | Threshold | Position | Score | Ubiquitinated |
| 44 | 0.75 | 0.30 | 44 | 0.47 | No |
| 46 | 0.37 | 0.30 | 46 | 0.46 | No |
| 127 | 0.90 | 0.30 | 77 | 0.66 | Yes, Low Confidence |
| - | - | - | 121 | 0.41 | No |
| - | - | - | 127 | 0.43 | No |
| - | - | - | 137 | 0.37 | No |
|  |  |  | 139 | 0.39 | No |
|  |  |  | 142 | 0.31 | No |
|  |  |  | 152 | 0.60 | No |

| **Table S6. RMSD value and TM-Score of all deleterious nsSNPs** | | | |
| --- | --- | --- | --- |
| **SNP ID** | **Residue change** | **RMSD value** | **TM-Score** |
| rs759413482 | V40M | 2.222 | 0.714 |
| rs1378953136 | R57L | 0.001 | 1 |
| rs753385457 | C61Y | 2.222 | 0.714 |
| rs1234813494 | G98R | 1.880 | 0.753 |
| rs760745635 | G108S | 2.173 | 0.709 |
| rs936261369 | D114N | 0.001 | 1 |
| rs1316376447 | E129G | 0.001 | 1 |
| rs1295542567 | A131T | 2.143 | 0.710 |
| rs373399618 | A131V | 0.001 | 1 |
| rs757003466 | I145T | 2.514 | 0.713 |

| **Table S7. ERRAT scores of each predicted model for selected mutant proteins** | | | | | | | | | | |
| --- | --- | --- | --- | --- | --- | --- | --- | --- | --- | --- |
| **Residue Change** | **Model 1** | | **Model 2** | | **Model 3** | | **Model 4** | | **Model 5** | |
|  | **C-Score** | **ERRAT-Score** | **C-Score** | **ERRAT-Score** | **C-Score** | **ERRAT-Score** | **C-Score** | **ERRAT-Score** | **C-Score** | **ERRAT-Score** |
| **V40M** | -2.36 | 73.154 | -2.82 | 78.52 | -3.47 | 64.42 | -3.52 | 72.48 | -3.82 | 40.26 |
| **C61Y** | -2.28 | 79.86 | -3.32 | 65.30 | -3.36 | 59.73 | -3.45 | 61.07 | -3.75 | 79.19 |
| **G98R** | -1.46 | 73.82 | -2.87 | 83.89 | -3.37 | 39.43 | -3.72 | 77.18 | -4.24 | 75.16 |
| **G108S** | -1.25 | 67.78 | -2.82 | 76.51 | -3.30 | 48.32 | -3.68 | 73.82 | -4.10 | 65.77 |
| **A131T** | -2.66 | 75.83 | -3.38 | 73.82 | -3.45 | 71.81 | 43.62 | -3.64 | -3.12 | 81.87 |

| **Table S8. Ramachandran plot statistics** | | | | |
| --- | --- | --- | --- | --- |
| **Variant** | **Most favoured regions** | **Additional allowed regions** | **Generously allowed regions** | **Disallowed regions** |
| Wild | 44.40% | 45.90% | 8.10% | 1.50% |
| V40M | 48.90% | 40.00% | 8.10% | 3.00% |
| G108S | 50.00% | 40.40% | 6.60% | 2.90% |
| G98R | 53.70% | 36.80% | 7.40% | 2.20% |
| C61Y | 47.40% | 40.70% | 8.10% | 3.70% |
| A131T | 40.00% | 45.90% | 8.90% | 5.20% |

| **Table S9. Secondary structure elements of wind and mutant variants** | | | | | | |
| --- | --- | --- | --- | --- | --- | --- |
| **Secondary structure** | **Wild** | **V40M** | **G108S** | **G98R** | **C61Y** | **A131T** |
| **Turn** | 83 | 88 | 87 | 82 | 81 | 88 |
| **Coil** | 25 | 25 | 38 | 18 | 24 | 31 |
| **Strand** | 33 | 30 | 19 | 43 | 37 | 32 |
| **3-10 Helix** | 6 | 3 | 3 | 6 | 6 | 3 |
| **Pi-Helix** | 5 | 0 | 0 | 0 | 0 | 0 |
| **AlphaHelix** | 0 | 7 | 6 | 7 | 4 | 0 |
| **Bridge** | 5 | 4 | 4 | 1 | 5 | 3 |

| **Table S10. Functional protein partners predicted by STRING** | | | | | |
| --- | --- | --- | --- | --- | --- |
| **S.NO** | **Functional Partners** | **Coexpression** | **Experiments** | **Textmining** | **Score** |
| 1 | WDR26 | Yes | Yes | Yes | 0.716 |
| 2 | ASPHD1 | No | No | Yes | 0.714 |
| 3 | HIRIP3 | No | No | Yes | 0.694 |
| 4 | FBXL2 | Yes | Yes | Yes | 0.69 |
| 5 | FAM57B | No | No | Yes | 0.683 |
| 6 | GDPD3 | Yes | No | Yes | 0.679 |
| 7 | C16orF92 | No | No | Yes | 0.669 |
| 8 | DOC2A | No | No | Yes | 0.639 |
| 9 | FBXL20 | Yes | Yes | Yes | 0.631 |
| 10 | KCTD13 | Yes | No | Yes | 0.62 |

| **Table S11. Interaction of YPEL3 with other genes and their network group.** | | | |
| --- | --- | --- | --- |
| **Gene 1** | **Gene 2** | **Weight** | **Network group** |
| KLHL24 | YPEL3 | 0.016014574 | Co-expression |
| PBXIP1 | YPEL3 | 0.010606633 | Co-expression |
| FCHO2 | YPEL3 | 0.0189282 | Co-expression |
| PPM1M | YPEL3 | 0.009881767 | Co-expression |
| ZC3H6 | YPEL3 | 0.01788707 | Co-expression |
| WIPI2 | YPEL3 | 0.010807981 | Co-expression |
| PPM1M | YPEL3 | 0.01020572 | Co-expression |
| KLHL24 | YPEL3 | 0.016959202 | Co-expression |
| WIPI2 | YPEL3 | 0.018653562 | Co-expression |
| PBXIP1 | YPEL3 | 0.016466597 | Co-expression |
| PPM1M | YPEL3 | 0.015632484 | Co-expression |
| YPEL5 | YPEL3 | 0.012987583 | Co-expression |
| KLHL24 | YPEL3 | 0.0135853 | Co-expression |
| WIPI2 | YPEL3 | 0.01884009 | Co-expression |
| PBXIP1 | YPEL3 | 0.018707583 | Co-expression |
| ZC3H6 | YPEL3 | 0.015187124 | Co-expression |
| YPEL4 | YPEL3 | 0.01283653 | Co-expression |
| FCHO2 | YPEL3 | 0.018167403 | Co-expression |
| CRBN | YPEL3 | 0.014361094 | Co-expression |
| LY6G6D | YPEL3 | 0.026688877 | Co-localization |
| LIN7A | YPEL3 | 0.025822364 | Co-localization |
| PDE4C | YPEL3 | 0.024180656 | Co-localization |
| UPK3B | YPEL3 | 0.023420567 | Co-localization |
| SLC9A5 | YPEL3 | 0.02275646 | Co-localization |
| PISD | YPEL3 | 0.022216145 | Co-localization |
| DOK7 | YPEL3 | 0.57323563 | Physical Interactions |
| YPEL4 | YPEL3 | 0.1535421 | Shared protein domains |
| YPEL5 | YPEL3 | 0.1535421 | Shared protein domains |
| YPEL2 | YPEL3 | 0.1535421 | Shared protein domains |
| YPEL1 | YPEL3 | 0.1535421 | Shared protein domains |
| MIS18A | YPEL3 | 0.1535421 | Shared protein domains |
| OIP5 | YPEL3 | 0.1535421 | Shared protein domains |
| CRBN | YPEL3 | 0.0728953 | Shared protein domains |
| YPEL4 | YPEL3 | 0.14810152 | Shared protein domains |
| YPEL5 | YPEL3 | 0.14810152 | Shared protein domains |
| YPEL2 | YPEL3 | 0.14810152 | Shared protein domains |
| YPEL1 | YPEL3 | 0.14810152 | Shared protein domains |
| MIS18A | YPEL3 | 0.14810152 | Shared protein domains |
| OIP5 | YPEL3 | 0.14810152 | Shared protein domains |
| CRBN | YPEL3 | 0.104303725 | Shared protein domains |

| **Table S12. Average value of simulation parameters for wild and mutant variants** | | | | | | |
| --- | --- | --- | --- | --- | --- | --- |
| **Parameters** | **WILD** | **V40M** | **G108S** | **G98R** | **C61Y** | **A131T** |
| RMSD | 0.44 | 0.56 | 0.61 | 0.48 | 0.40 | 0.37 |
| RMSF | 0.17 | 0.24 | 0.22 | 0.21 | 0.19 | 0.19 |
| Gyrate | 1.64 | 1.55 | 1.65 | 1.60 | 1.61 | 1.58 |
| SASA | 98.39 | 93.37 | 101.44 | 96.94 | 101.35 | 92.73 |
| H-Bond | 76 | 84 | 76 | 75 | 72 | 76 |

**List of Supplementary figures**

**
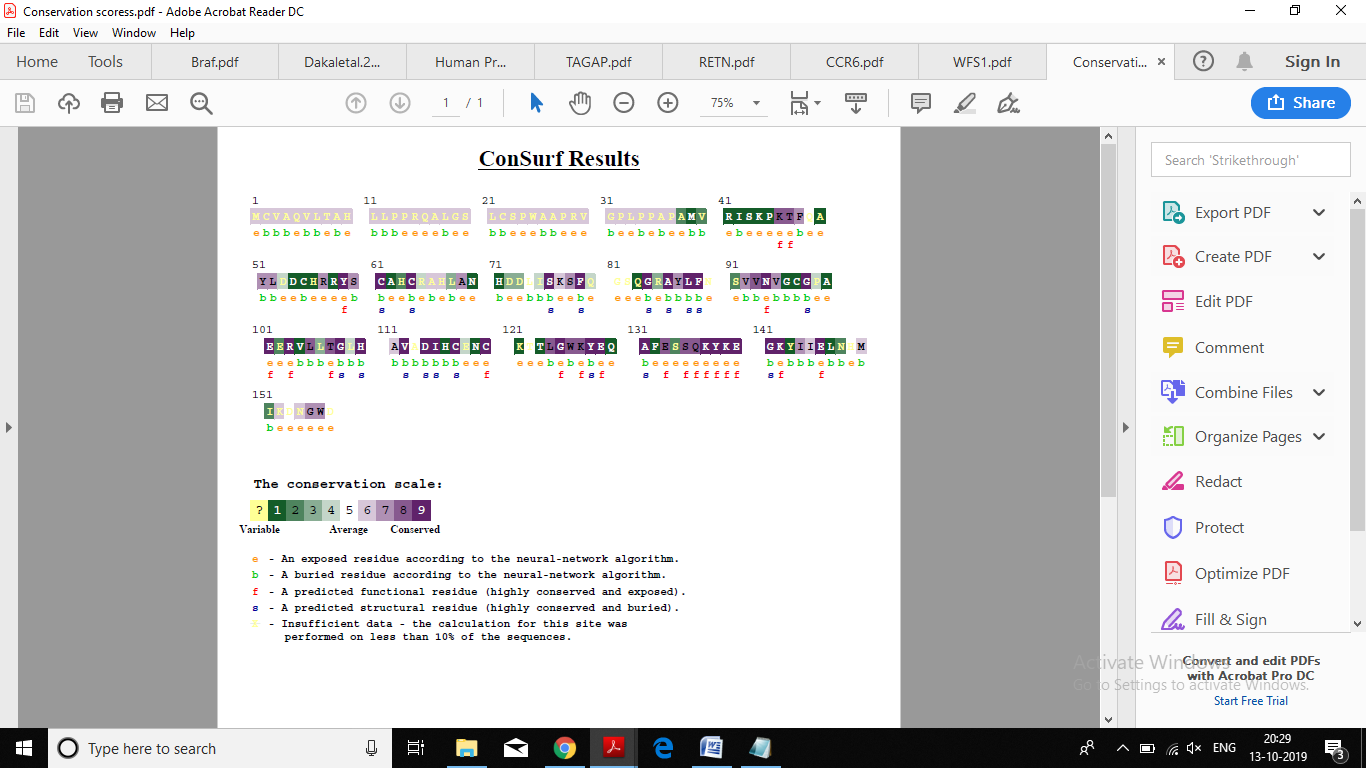
**

Figure S1. Conservation scores of each amino acid residue of YPEL3 protein

Figure S2. Global quality of predicted protein models analyzed by ProSA. A) Wild, B) V40M, C) G108S, D) G98R, E) C61Y, F) A131T.

Figure S3. Representation of secondary structure elements of wild and mutant protein variants.


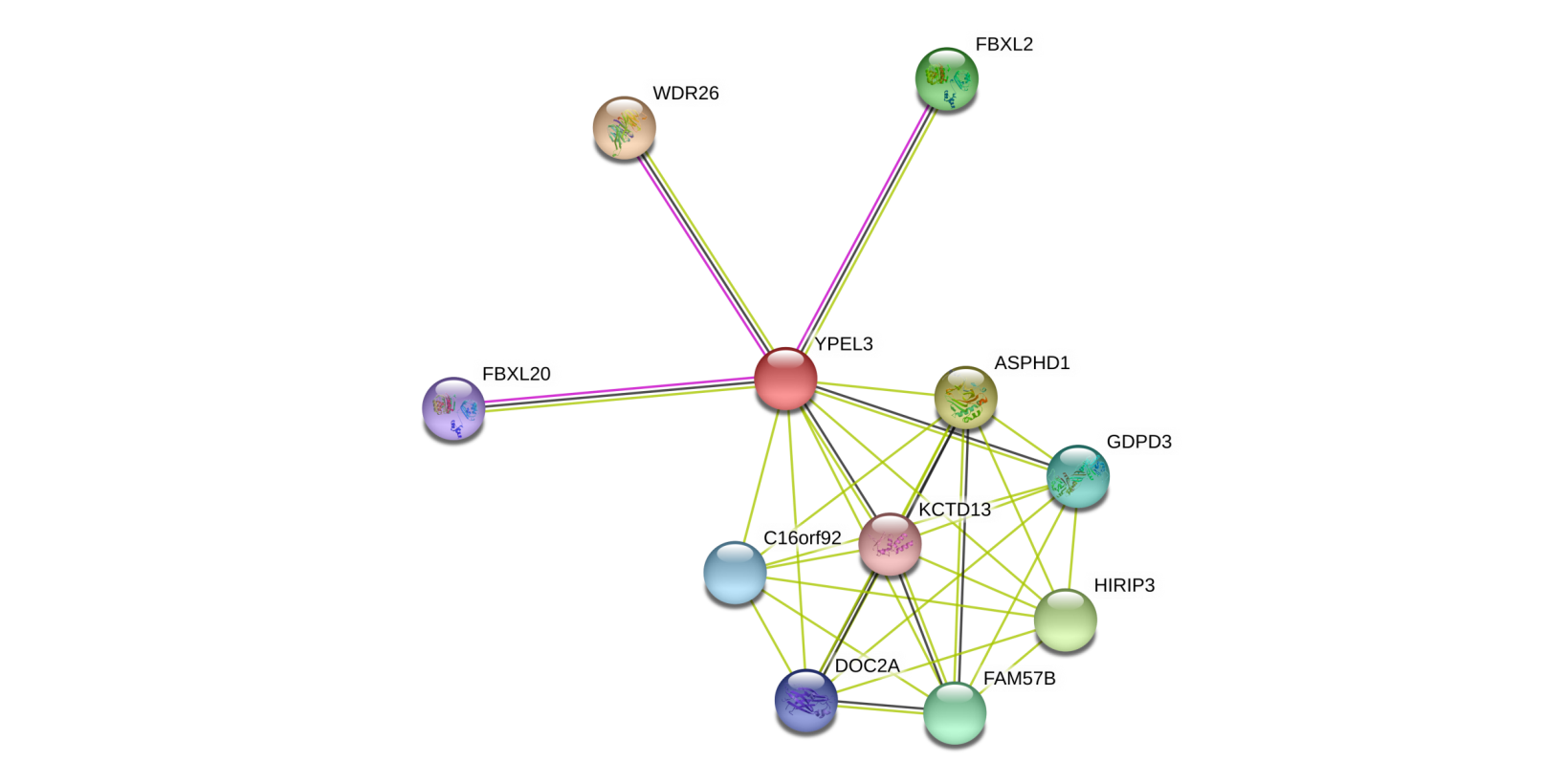


Figure S4. YPEL3 interaction network with other genes analyzed by STRING.

Figure S5. Functional interaction network analysis of YPEL3 observed by GeneMANIA database.

Figure S6. Molecular dynamic simulation: RMSD analysis of wild (Black) with the mutant variants (Red) A) V40M, B) G108S, C) G98R, D) C61Y, E) A131T.

Figure S7. Molecular dynamic simulation: RMSF analysis of wild (Black) with the mutant variants (Red) A) V40M, B) G108S, C) G98R, D) C61Y, E) A131T .

Figure S8. Molecular dynamic simulation: Radius of Gyration analysis of wild (Black) with the mutant variants (Red) A) V40M, B) G108S, C) G98R, D) C61Y, E) A131T.

Figure S9. Molecular dynamic simulation: Hydrogen Bond analysis of wild (Black) with the mutant variants (Red) A) V40M, B) G108S, C) G98R, D) C61Y, E) A131T.

Figure S10. Molecular dynamic simulation: SASA analysis of wild (Black) with the mutant variants (Red) A) V40M, B) G108S, C) G98R, D) C61Y, E) A131T.
